# Adrenergic Modulation of Cortical Gain and Sensory Processing in the Mouse Visual Cortex

**DOI:** 10.3390/brainsci15040406

**Published:** 2025-04-17

**Authors:** Ricardo Medina-Coss y León, Elí Lezama, Inmaculada Márquez, Mario Treviño

**Affiliations:** 1 Laboratorio de Plasticidad Cortical y Aprendizaje Perceptual, Instituto de Neurociencias, Universidad de Guadalajara, Guadalajara 44130, Jalisco, Mexico; 2 School of Medicine, Southern Illinois University, Carbondale, IL 62901, USA; 3 Departamento de Ciencias Médicas y de la Vida, Centro Universitario de la Ciénega, Universidad de Guadalajara, Ocotlán 47820, Jalisco, Mexico; 4 Departamento de Psicología, Centro Universitario de la Ciénega, Universidad de Guadalajara, Ocotlán 47820, Jalisco, Mexico

**Keywords:** neuronal variability, norepinephrine, visual processing, cortical oscillations, sensory noise

## Abstract

**Background/Objectives:** Sensory perception is influenced by internal neuronal variability and external noise. Neuromodulators such as norepinephrine (NE) regulate this variability by modulating excitation–inhibition balance, oscillatory dynamics, and interlaminar connectivity. While NE is known to modulate cortical gain, it remains unclear how it shapes sensory processing under noisy conditions. This study investigates how adrenergic modulation affects signal-to-noise processing and perceptual decision-making in the primary visual cortex (V1) of mice exposed to varying levels of visual noise. **Methods:** We performed in vivo local field potential (LFP) recordings from layers 2/3 and 4 of V1 in sedated mice to assess the impact of visual noise and systemic administration of atomoxetine, a NE reuptake inhibitor, on cortical signal processing. In a separate group of freely moving mice, we used a two-alternative forced-choice to evaluate the behavioral effects of systemic and intracortical adrenergic manipulations on visual discrimination. **Results:** Moderate visual noise enhanced cortical signal processing and visual choices, consistent with stochastic resonance. High noise levels impaired both. Systemic atomoxetine administration flattened the cortical signal-to-noise ratio function, suggesting disrupted gain control. Behaviorally, clonidine impaired accuracy at moderate noise levels, while atomoxetine reduced discrimination performance and increased response variability. Intracortical NE infusions produced similar effects. **Conclusions:** Our findings demonstrate that NE regulates the balance between signal amplification and noise suppression in a noise- and context-dependent manner. These results extend existing models of neuromodulatory function by linking interlaminar communication and cortical variability to perceptual decision-making.

## 1. Introduction

Sensory perception is inherently variable, shaped by external and internal noise sources. External noise, such as fluctuations in stimulus contrast and internal noise arising from spontaneous neural activity and synaptic variability, influence perceptual accuracy and decision-making [1,2,3]. While noise is often considered detrimental to signal detection, moderate noise levels can paradoxically enhance perception under certain conditions through stochastic resonance (SR)-like phenomena. SR occurs when noise amplifies weak signals, enhancing sensory processing; however, at higher levels, noise dominates the signal and impairs performance [4,5].

The primary visual cortex (V1) is crucial in integrating feedforward thalamocortical inputs with local recurrent processing to encode visual stimuli. Neuronal activity in V1 is strongly influenced by contrast-dependent mechanisms that modulate neural gain, selectivity, and response reliability. Neural gain refers to how the input–output balance of neurons is adjusted, allowing for adaptive responses to varying inputs. This modulation is mediated by GABAergic inhibition, synaptic fluctuations, neuromodulatory input, and shifts in cellular conductance [6,7]. Contrast responses in V1 also depend on stimulus size and receptive field integration. While increasing stimulus contrast generally enhances V1 responses, larger stimuli that extend beyond individual receptive fields introduce competitive interactions between central and peripheral visual regions, leading to surround suppression effects [8,9]. Studies in primates and rodents show that contrast modulates local field potential (LFP) activity, particularly in the gamma band, where broadband gamma power increases while narrowband gamma oscillations decrease with rising contrast [10,11,12]. These findings suggest that contrast influences V1 excitability and recurrent interactions, shaping visual processing across cortical layers.

A fundamental feature of cortical circuits is their ability to balance signal amplification and noise suppression. At low contrast, lateral intracortical interactions integrate visual inputs, but as contrast increases, feedforward thalamocortical input dominates, reducing the influence of lateral connectivity [13]. One proposal is that neuromodulators such as norepinephrine (NE) and acetylcholine (ACh) fine-tune cortical network dynamics by modulating neural variability, thereby enabling circuits to remain flexible while preserving the stability of sensory representations [14,15]. NE, released from the locus coeruleus (LC), plays a well-established role in regulating cortical excitability, enhancing the signal-to-noise ratio (SNR), and supporting attentional processes and perceptual learning [16,17,18]. In V1, NE influences stimulus encoding by modulating neural gain and refining contrast sensitivity, contributing to the optimization of visual responses under varying stimulus conditions [19]. However, despite this evidence, it remains unclear as to how NE influences the cortical processing of visual stimuli embedded in external noise—a crucial gap in our knowledge of how neuromodulatory systems influence perception under more naturalistic, noisy conditions.

Contrary to earlier findings suggesting that NE enhances sensory responses, our previous research showed that adrenergic receptor activation in mouse V1—mainly through α_1_- and β-adrenergic receptors—induces divisive gain control, reducing overall cortical activity without affecting contrast sensitivity (or orientation tuning) [19]. This divisive effect impairs discrimination performance. Additionally, micro-infusions of adrenergic receptor agonists into V1 increase choice variability, particularly at high contrast and low spatial frequencies, suggesting that adrenergic modulation disrupts perceptual stability under specific luminance conditions [19]. To investigate how adrenergic modulation influences sensory processing under varying noise conditions, we conducted two separate experiments using different groups of mice. In the first, we performed in vivo local field potential (LFP) recordings from layers 2/3 (L2/3) and 4 (L4) of the primary visual cortex (V1) in sedated animals to characterize cortical responses to stimulus and noise contrast and to determine whether the signal-to-noise ratio (SNR) followed a nonlinear profile consistent with stochastic resonance (SR) theory. In the second, we evaluated the behavioral effects of adrenergic modulation in freely moving mice using a two-alternative forced-choice (2AFC) visual discrimination task in a hexagonal water maze, measuring performance under varying levels of visual noise and pharmacological manipulation of norepinephrine (NE) signaling.

## 2. Materials and Methods

### 2.1. Animals

Two–four month-old C57BL/6J male mice (weight 25 g ± 7 g) were housed in conventional polycarbonate cages under regular laboratory conditions, with unlimited access to food (Rodent Lab Chow 5001, Purina, Jalisco, Mexico) and water (groups of no more than three mice per cage). The housing room featured a 12:12 h light–dark cycle, with a constant room temperature and humidity (22 °C ± 2 °C). All tests followed the Mexican animal welfare guidelines (SAGARPA, NOM-062-ZOO-1999) and the National Institutes of Health’s Guide for the Care and Use of Laboratory Animals. The experimental protocols utilized in this study were approved by our institution’s ethical committee (Instituto de Neurociencias, Universidad de Guadalajara, México: ET122023-383).

### 2.2. Surgical Procedures

We used conventional surgical techniques to implant electrodes or cannulae into the mouse’s primary visual cortex (V1; [19,20]). To sedate the animals, we used a mixture of fentanyl (Fenodid, 0.15 mg/kg *i.p.*; Laboratorios Pisa, Jalisco, Mexico), midazolam (Dormicum, 6 mg/kg *i.p.*; Laboratorios Pisa), and dexmedetomidine (Dexdomitor, 0.5 mg/kg *i.p.*; Orion Pharma, Zoetis, Ciudad de Mexico, Mexico). We then covered their eyes with ophthalmic lubricant and subcutaneously injected small amounts of lidocaine (Piscana 2%; Laboratorios Pisa) at the incision zones. We then fixed the mice in a stereotaxic apparatus (Stoelting Co., Wood Dale, IL, USA; Model Nr.: 51730) and controlled their body temperature (36.5–38 °C) with a heating blanket with a feedback circuit (Homeothermic Monitor; Harvard Apparatus, Cambridge, MA, USA, Model 50-7212). After making an incision in the skin over the head, we deflected the scalp and removed any tissue from the bone. We then marked the coordinates targeting V1 (−4.29 mm AP, ±2.75 mm ML, 0.6 mm DV from the dura; [20,21]) and drilled the bone using a 0.8 mm dental drill (Dremel 105; Foredom, MH-170). Craniotomies were unilateral for electrode implantation and bilateral for cannulae implantation. We carefully removed the dura using a curved-tip syringe and, with the aid of the stereotaxic apparatus, positioned either a microdrive (Harlan 4 Drive, Neuralynx, MT, USA) for electrophysiological recordings, or stainless steel cannulae (30-gauge, BD Precision Glide^TM^ Needles) for micro-infusion experiments [19,20]. For electrophysiology, we secured the microdrive with two hex-head anchor screws, ensuring the dura was in contact with the ground screws. Copper wires, pre-soldered to the screw heads, were connected to additional cables from the microdrive. These were routed from the drive’s lower cone to the screws, with excess wire wrapped around the adjacent screws in a back-and-forth pattern. This dual-ground configuration provided stable electrical reference points while maintaining mechanical integrity for chronic LFP recordings in V1. For micro-infusion experiments, we implanted stainless steel cannulae at the target coordinates. Both microdrives and cannulae were secured to the scalp using dental acrylic cement (Nic-Tone R6V, MDC Dental, Jalisco, Mexico) to ensure long-term stability. Once the cement had hardened on the microdrives, we carefully lowered the electrodes to position them within L2/3 (at a depth of 350–500 μm) and L4 (at a depth of 150–350 μm; see Section 2.4). For the cannulae, we inserted stainless steel obturators to prevent clogging. After surgeries, the mice were individually housed and kept under vigilance until they recovered (~5 days) before initiating experiments.

### 2.3. Visual Stimuli

We created and utilized three primary types of visual stimuli, designated as follows: grating-only [G], noise-only [N], and combined grating plus noise ([G + N]). The primary visual stimuli [G] were composed of horizontal or vertical sine-wave gratings with a spatial frequency of 0.04 cycles per degree. These stimuli were presented at two contrast levels, 0% and 12.5%, and were generated using the Psychophysics Toolbox extension (PTB-3) in MATLAB. In some cases, we incorporated precomputed visual noise composed of randomly generated squares with a spatial frequency of 0.2 cycles per degree. The size of these squares was chosen based on previous studies to ensure they were detectable and processed by the mouse visual system [22]. These squares displayed variable contrasts drawn from a uniform distribution, ensuring that the combination [G + N] produced images that did not saturate in white. The average [N] contrast levels were set at 0%, 5%, 7%, 9%, 11%, 30%, and 60%, informed by previous empirical measurements from our lab [5]. This former study revealed that intermediate levels of background noise contrast, specifically within the 7–11% range, enhanced motion discrimination performance. Additionally, we included higher noise levels (30% and 60%) to evaluate potential performance degradation. Visual noise was updated at 60 frames per second (FPS). To maintain a uniform average luminance across the screen, we structured the stimuli presentation as follows. The [N] stimuli consisted of a fixed grid of 10 × 8 squares per screen, ensuring that local contrast variations were evenly distributed. Similarly, the sine-wave gratings were presented at full-cycle spatial frequencies without unwanted spatial artifacts [23,24]. Furthermore, during the experiments, trials containing [G + N] were paired with [N]-only trials, with the order of presentation within each pair randomized to balance order effects.

### 2.4. Electrophysiological Experiments

We performed LFP recordings in V1 of head-fixed mice using custom-built microdrives. These microdrives contained eight tungsten electrodes (impedance: 0.2–5 MΩ; Ø = 0.06 mm; NanoZ, Neuralynx) with an insulating polyimide coating. Each electrode assembly was composed of paired tungsten wires to enhance stability during cortical insertion. In each electrode pair, one electrode was approximately 150 μm longer than the other. The electrodes were positioned across L2/3 and L4 of V1. This configuration allowed the recording of neural activity across these two cortical layers. Therefore, electrodes targeting L4 were implanted at 350–500 μm depth, while those targeting L2/3 were implanted at 150–350 μm depth. All recordings were performed using the Neuralynx acquisition system (Digital Lynx 4SX, Neuralynx, MT, USA), with impedance values verified before microdrive implantation using a NanoZ impedance testing device (Z = 3.83 ± 0.13 MΩ).

**Figure 1:**
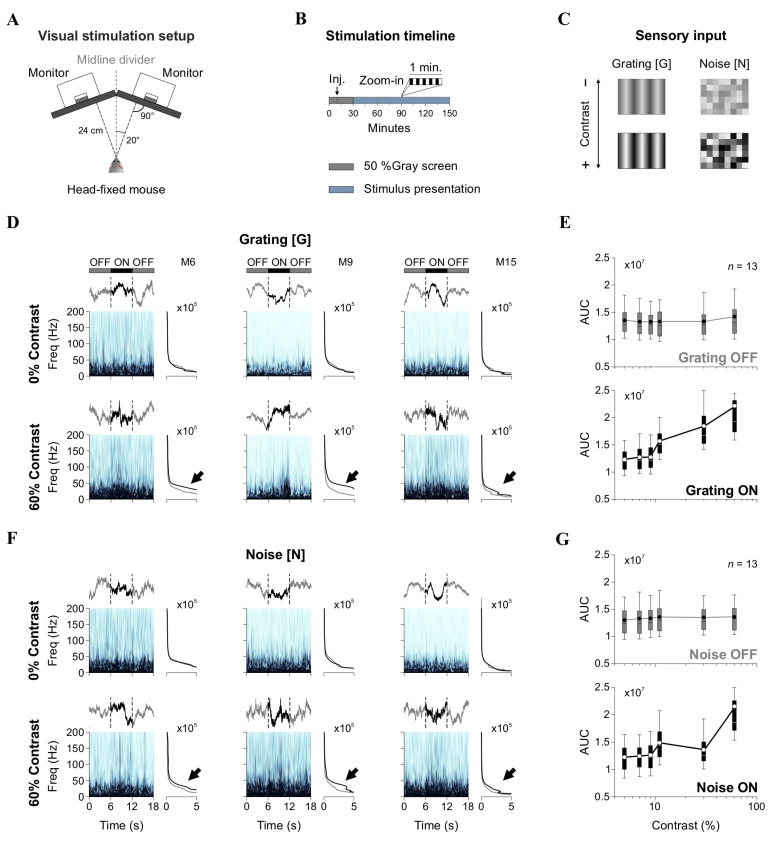
Experimental setup and spectral analysis of LFP responses. (**A**) Schematic of the visual stimulation setup. Two monitors positioned at an optimal tilt present visual stimuli while LFP recordings are conducted in head-fixed, sedated mice. (**B**) Stimulation timeline. Recordings began with a 10 min baseline (black screen), followed by 20 min of spontaneous activity post-drug administration. Visual stimulation lasted 120 min using a 6 s ON (stimulus)/6 s OFF (black screen) paradigm. (**C**) Visual stimuli consisted of gratings ([G]) and noise ([N]) at varying contrast levels. (**D**) Wavelet spectrograms from L2/3 LFPs showing spectral power dynamics in three sample mice (M6, M9, M15) during OFF (black screen) and ON (stimulus) epochs. Spectral power is represented using a light-to-dark blue colormap, where darker shades indicate higher power. Insets display the averaged spectrograms; gray traces represent the averages from the two flanking OFF epochs; and black traces represent the average from the 6-s ON epoch. Black arrows point to the spectral differences between OFF and ON epochs. (**E**) Group-averaged area under the curve (AUC) of spectrograms in OFF and ON epochs for [G]. (**F**,**G**) Equivalent analysis for [N] stimulation.

During the recordings, mice were lightly sedated to maintain a calm yet active cortical state, optimizing conditions for obtaining high-quality local field potential (LFP) recordings. The absence of pedal and tail-pinch reflexes confirmed the depth of sedation. During sedation, the mouse’s head was secured in a modified stereotaxic apparatus (generously provided by colleagues from the Max Planck Institute in Heidelberg, Germany). This setup was used without a nose holder to ensure the mouse’s vision was unobstructed during the procedure. The visual stimuli were presented on two contiguous monitors (1920 × 1080 pixels; 27-inch; Dell P2414H, Dell Technologies, Chongqing, Sichuan, China), positioned with an optimal tilt to maximize coverage of the mouse’s visual field [25] (Figure 1A). Synchronization between the projection of visual stimuli and LFP recordings was achieved using TTL signals integrated with the Neuralynx acquisition system.

**Figure 2:**
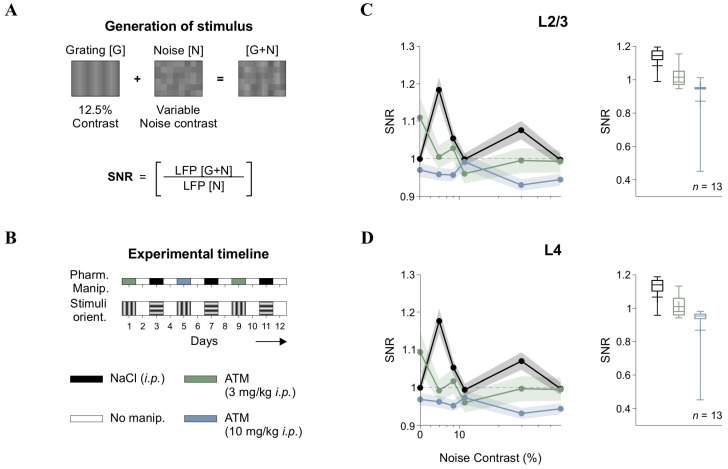
Signal-to-noise ratio (SNR) and atomoxetine effects. (**A**) Schematic of [G + N] stimulus generation via pixel-wise summation of gratings [G] and noise [N]. SNR was calculated by comparing the AUC of LFP wavelet power spectra during [G + N] epochs to noise-only ([N]) epochs. (**B**) Experimental timeline, ensuring drug washout and randomized stimulus orientation across sessions. Pharmacological conditions are represented by different colors, allowing comparisons in the following panels. (**C**) SNR in L2/3 across noise contrast levels under control and atomoxetine (ATM) conditions. (**D**) Equivalent analysis for L4 LFPs, showing ATM-induced SNR disruption. Horizontal axis (contrast) in panels **C** and **D** is in semilog scale.

LFP traces were recorded with a sampling rate of 8 kHz. They were then downsampled to 400 Hz to streamline processing time for subsequent spectral analysis. Baseline (control) recordings were collected for 10 min in the absence of visual stimulation (black screen), followed by an intraperitoneal injection of either atomoxetine (low dose: 3 mg/kg; or high dose: 10 mg/kg) or isotonic saline (NaCl 0.9%). After drug administration, we recorded 20 additional minutes of spontaneous LFP activity without visual stimulation. LFP responses to visual stimuli were then recorded for 120 min, using a 6 s ON/6 s OFF presentation paradigm (total of 650 cycles) (Figure 1B). During the OFF epochs, a black screen was displayed. During the ON epochs, [G], [N], or [G + N] stimuli could be projected. Saline drops were periodically applied to the eyes of the mice to maintain moisture and avoid dryness. To prevent drug carryover effects [19], we implemented a four-day washout period between intraperitoneal atomoxetine injections, as depicted in Figure 2B. We alternated the orientation of gratings (using orthogonal angles) across recording sessions to minimize neural adaptation and potential overexposure of V1 ensembles [26].

We analyzed our LFP data to quantify spectral power distributions using the one-dimensional Morlet wavelet transform [27]. We examined ON and OFF epochs separately, allowing us to assess how changes in [G + N] levels influenced LFP power spectra. To determine the impact of [N] on cortical activity, we calculated the SNR by comparing the area under the curve (AUC) of spectral power between the [G + N] and [N] conditions within the 5–50 Hz frequency range, following previous methodologies [28].

(1) $$SNR=\frac{AUC\{Power\;Spectrum\left[G+N\right]\}}{AUC\{Power\;Spectrum\left[N\right]\}}$$

In our figures, we normalized the SNR values to those obtained with vehicle injection (NaCl, *i.p.*) at [N] with 0% contrast, which corresponds to a 50% gray screen. Before normalization, the SNR values were greater than 1 for the [N] with 0% contrast condition.

### 2.5. Partial Directed Coherence

We employed partial directed coherence (PDC) to examine causal interactions between LFP signals from L4 and L2/3. To enhance our analysis, we utilized generalized orthogonalized partial directed coherence, specifically designed for nonstationary multichannel recordings [29]. Due to the computational intensity of the algorithm, we further downsampled the LFP data from 400 Hz to 100 Hz. We then selected three epochs within each recording session relative to the timing of stimulus presentation trials: early (t = 0 min), intermediate (t = 65 min), and late (t = 120 min) phases. During these periods, we combined LFP data from both OFF and ON epochs, enabling us to explore broader changes in cortical connectivity in response to adrenergic modulation. To validate our connectivity measures, we generated surrogate PDC values using the iterative amplitude adjusted Fourier transform (IAAFT) algorithm. This method produces surrogate signals that maintain the original signal’s amplitude spectrum and distribution while randomizing the phase, thereby disrupting the temporal structure. By matching the original amplitude and rank-order distribution, IAAFT surrogates test the null hypothesis that observed PDC values arise from linear, stationary processes. This approach helped us distinguish genuine directed interactions from spurious effects caused by shared noise, nonstationarities, or nonlinearities. We present the PDC estimations using spectrogram-like plots, which allow for comparison between original connectivity measures and surrogate-based coherence values.

### 2.6. Behavioral Experiments

We tested a separate cohort of mice in a two-alternative forced-choice (2AFC) visual discrimination task, described previously [20]. Experiments were conducted from 8 a.m. to 2 p.m., Monday through Friday, with groups of 10 mice. Each session comprised up to 70 daily trials and lasted approximately one hour. Mice underwent a 15–20-day training program in which perceptual and physical performance gradually increased from 10 to 70 daily trials. Pharmacological treatments, including intraperitoneal (*i.p.*) and intracortical (*i.c.*) drug administrations, were performed 10 min before initiating the task, following a previously validated protocol [21].

For *i.p.* injections, we administered one of the following: saline (NaCl), clonidine (an α_2_-adrenergic agonist, 10 mM), or atomoxetine (a norepinephrine reuptake inhibitor, 10 mg/kg), each dissolved in a vehicle solution (NaCl 0.9%). Injections were performed in the right caudal quadrant of the abdomen. For *i.c.* injections, mice with pre-implanted bilateral cannulae targeting V1 received infusions through injectors connected to 10 μL Hamilton syringes driven by a custom-built micro-infusion pump [20]. The drugs used for cortical micro-infusions included norepinephrine (NE: 0.025 mM or 100 mM), or a mixture of adrenergic antagonists (prazosin, α_1_-AR antagonist, 10 mM; yohimbine, α_2_-AR antagonist, 10 mM; propranolol, β-AR antagonist, 10 mM), dissolved in vehicle solution (NaCl 0.9%). The selection of 0.025 mM and 100 mM NE was guided by a dose–response relationship reported previously [19], where lower concentrations produced sub-maximal effects (~30% reduction in visual discrimination performance), while higher concentrations induced near-maximal suppression (~75% reduction). Physiological NE levels in synapses typically range from 3 to 5 µM; however, micro-infused drugs rapidly diffuse and dilute in the extracellular space, creating a steep concentration gradient. Thus, to achieve transiently effective concentrations in the low µM range at the target site, an initial infusion concentration of 100 mM is necessary. In contrast, lower doses (e.g., 0.025 mM) may only briefly reach threshold levels, accounting for their sub-maximal effects. The total infusion volume per hemisphere was 500 nL at a constant rate of 0.1 μL/min (1.67 nL/s). The presence of an air pocket in the tubing allowed us to verify drug delivery. Throughout and after the injections, no signs of discomfort were observed in the mice. All drugs were purchased from Sigma-Aldrich and freshly prepared before each infusion [19].

During testing (with only one mouse/pool at a time), the experimenter stayed out of the animal’s field of view to minimize any interference with its behavior [20]. At the end of each session, mice were carefully dried with a towel and returned to their home cage. The testing environment was meticulously controlled to eliminate distractions for mice (and experimenters). All trials were conducted in complete silence, with minimized external scents and no use of electronic devices. We measured the percentage of correct choices across conditions to assess visual discrimination performance. We recorded the swimming paths of the mice (LifeCam Studio; 30 FPS, Microsoft Corporation, Guangdong, China) and quantified key behavioral metrics, including path length (in cm) and escape latency (time to reach and climb the platform, in s) [20,24]. As part of our procedures, we varied the [N] contrast in the visual stimuli, using 0%, 7%, 12%, 20%, and 30% levels. To quantify the influence of [N] on discrimination performance, we paired trials containing [G + N] with trials containing [G] only and computed a Correct Choice Index (CCI; [5]):

(2) $$CCI=\frac{\%Correct\left[G+N\right]}{\%Correct\left[G\right]}$$

and the escape latency index (ELI):

(3) $$ELI=\frac{EL\left[G+N\right]}{EL\left[G\right]}.$$

We approximated the CCI (and the escape latency index, ELI) responses as a function of [N] contrast (c) by linearly adding a logistic curve with a Gaussian distribution:

(4) $$CCI=\left\{\frac{L}{1+e^{k\left(c_{i}-c^{*}\right)}}\right\}+\left\{Amp\cdot e^{\left(\frac{-{\left(c_{i}-\mu \right)}^{2}}{2\sigma^{2}}\right)}\right\}$$

where L is the maximum value of the logistic function, k is the slope of the curve, c* is the x-value of the sigmoid’s midpoint, and Amp is the amplitude of the Gaussian with μ mean and σ standard deviation [5]. All data analyses were performed in MATLAB R2023 (MathWorks, Inc.; Natick, MA, USA).

### 2.7. Histology

Following the completion of the experiments, we performed histological analyses to confirm the precise placement of electrodes and cannulae. Mice were euthanized using a single dose of sodium pentobarbital (100–150 mg/kg *i.p.*; Pisabental; Laboratorios Pisa), followed by transcardial perfusion with 4% paraformaldehyde in 0.9% saline. We induced in vivo electrolytic lesions in animals with implanted electrodes through the recording electrodes to facilitate localization. After extracting the brains, we photographed them using a Zeiss (Jena, Germany) Stemi 305 stereomicroscope equipped with a universal mount. These images were then aligned with a mouse brain atlas to verify the accurate placement of the electrodes or cannulae [20].

### 2.8. Statistical Analysis

We employed ANOVA and repeated-measures ANOVA (RM-ANOVA) to compare experimental conditions. When normality was violated, we used the Friedman test as an alternative to RM-ANOVA and reported the Greenhouse–Geisser corrected epsilon (ε) values. Kruskal–Wallis (KW) tests were applied, followed by Bonferroni-corrected *post hoc* comparisons when necessary. All data are presented as mean ± SEM, with statistical significance set at *p* < 0.05.

## 3. Results

### 3.1. Sensitivity of LFP Power to Grating and Noise Contrast in V1

Previous studies in rodents and primates show that increasing stimulus contrast leads to spectral changes in V1. Specifically, broadband gamma power in the upper layers increases, suggesting that higher contrast levels enhance excitatory and inhibitory interactions within the cortical circuitry [10,11,12,13]. To confirm the impact of stimulus contrast on neural activity in V1, we recorded LFPs in mice while projecting visual stimuli. Our experimental setup consisted of two monitors arranged with a tilted angle to maximize coverage of the mouse’s visual field [25] (Figure 1A). Mice were lightly sedated to maintain stable recording conditions while preserving strong cortical responsiveness. For all sessions, we first collected baseline recordings for 30 min without visual stimulation (monitors projecting a black screen). We then recorded LFP responses to visual stimuli for 120 min using 6 s ON/6 s OFF presentation cycles (total of 650 repetitions) (Figure 1B). During the OFF periods, black screens were shown. We recorded LFP signals from tungsten electrodes targeting L4 and L2/3 of V1. These layers were chosen due to their crucial roles in processing visual information: L4 receives direct thalamic input and is primarily involved in feedforward sensory processing, while L2/3 integrates lateral and top-down inputs, playing a role in contextual modulation and higher-order processing [10,11].

The visual stimuli consisted of sinusoidal gratings ([G]) at varying contrast levels and randomized noise patterns ([N]) composed of visible squares with fluctuating contrast values [22] (Figure 1C). We analyzed the spectral power of LFP responses by extracting epochs corresponding to [G] and [N] presentations. Figure 1D shows representative wavelet power spectra from L2/3 of V1 from three sample mice, illustrating both inter-individual variability and consistent stimulus-driven (spectral) modulations. Notably, although single evoked potentials lacked evident time-locked components, the spectrograms revealed reliable changes in power across animals in response to visual stimulation. More specifically, the spectrograms showed increases in power within the 5–50 Hz range during [G] stimuli at 60% contrast compared to 0%, as indicated by black arrows in the right insets. Quantitative analysis confirmed that stimulus contrast enhanced the LFP power area under the curve (AUC, repeated-measures ANOVA, *F*_5,1225_ = 4.27, *p* < 0.001; Friedman test, χ^2^_245_ = 1200, *p* < 0.001, *n* = 13), with *post hoc* comparisons (Durbin–Conover) revealing differences between OFF and ON epochs (Figure 1E). Similarly, [N] stimuli produced contrast-dependent increases in LFP power (RM ANOVA, *F*_5,1225_ = 2.23, *p* = 0.04; Friedman test, χ^2^_245_ = 849, *p* < 0.001, *n* = 13) (Figure 1F,G), indicating that both structured and noisy inputs modulate cortical activity in L2/3 LFPs of V1.

### 3.2. Atomoxetine Disrupts the SNR Response Function in the Visual Cortex

In cortical responses, “signal” refers to neural activity that conveys meaningful sensory information, such as responses to specific stimuli ([G]), while “noise” pertains to background neural activity lacking relevant information and potentially obscuring signal detection [30]. Sensory systems are thought to encode information by optimizing the SNR, enhancing the signal while minimizing noise to improve perceptual accuracy. In V1, SNR is thought to determine the fidelity of sensory representations, with variations in contrast influencing how external noise interacts with cortical processing. To investigate how [N] contrast influenced neural responses, we calculated SNR from LFPs recorded in L2/3 and L4 of V1. The SNR was defined as the ratio of the area under the curve (AUC) of the wavelet power spectra for visually evoked LFPs during the linear summation of low-contrast gratings plus noise ([G + N]) compared to the background activity recorded during noise-only ([N]) epochs (Figure 2A). Experimental sessions were conducted over multiple days, as we tested various pharmacological treatments on the same mice using a procedure we previously validated [19]. Furthermore, we varied grating orientations across recording sessions to prevent overexposure [26] and maintained a four-day interval between drug injections to ensure adequate drug washout (Figure 2B).

Analysis of group-averaged SNR in L2/3 showed that the response pattern consistently remained above one, with low contrast levels achieving peak SNR values. This suggests optimal signal amplification at these contrast levels, before stabilizing or slightly decreasing at other levels in L2/3 (*F*_5,80_ = 10.1, *p* < 0.001, *η*^2^_G_ = 0.183, *n* = 13; black trace in Figure 2C) and L4 (*F*_5,80_ = 9.69, *p* < 0.001, *η*^2^_G_ = 0.179, *n* = 13; black trace in Figure 2D). Therefore, this pattern suggests that moderate levels of visual [N] enhanced cortical responses, a phenomenon consistent with stochastic resonance (SR) [13,31,32]. Since the SNR function was not flat but instead showed an optimal [N] level for maximizing output, we hypothesized that internal [N] sources, like those influenced by varying levels of neuromodulation, could transform the SNR curve. To test this hypothesis, we administered intraperitoneal (*i.p*.) injections of atomoxetine (ATM), a norepinephrine reuptake inhibitor, at low (3 mg/kg) and high (10 mg/kg) doses. This approach aimed to determine whether adrenergic signaling could modulate the observed cortical SNR function.

We found that ATM administration disrupted the SNR function in a dose-dependent manner (Figure 2C,D). Both low and high doses of ATM disrupted the SNR pattern observed under control conditions, diminishing the peak SNR values achieved at low contrast levels, leading to a reduction in SNR across all noise contrast levels in L2/3 and L4 (Friedman test, χ^2^_17_ = 58, *p* < 0.001 for L2/3; χ^2^_17_ = 44.6, *p* < 0.001 for L4). Pairwise comparisons revealed differences between both ATM conditions and controls across nearly all contrasts, except for very low (e.g., 11%) and high contrast levels (e.g., 60%).

The disruption of the cortical SNR function by ATM implies that norepinephrine may have shifted the balance between excitatory and inhibitory activity towards increased inhibition [19]. Other studies have indicated that neuromodulators can affect SNR through divisive gain control mechanisms, which adjust the response gain of neurons relative to stimulus contrast [15,33]. Noradrenergic activity has also been implicated in state-dependent modulation of cortical responses, with effects that vary across layers and behavioral conditions [34,35].

### 3.3. Atomoxetine Disrupts Interlaminar Communication in the Visual Cortex

V1 is organized into six layers that interact through a hierarchical processing of sensory information. L4 is the principal recipient of thalamic input from the dorsal lateral geniculate nucleus (dLGN), transmitting processed signals to L2/3. This feedforward flow plays a crucial role in visual encoding, but weaker feedback projections from L2/3 to L4 also contribute to recurrent processing and gain modulation. To investigate the impact of adrenergic neuromodulation on interlaminar interactions, we analyzed directional influences between simultaneously recorded LFPs from L4 and L2/3 using Partial Directed Coherence (PDC).

**Figure 3:**
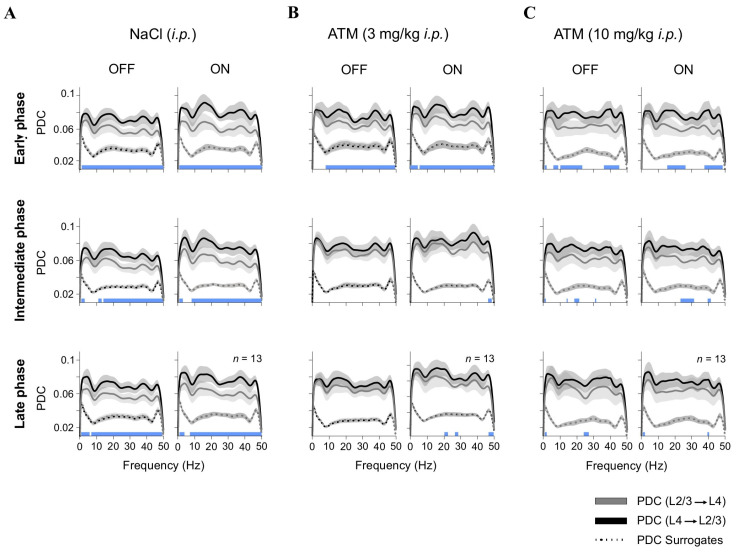
Adrenergic modulation of interlaminar connectivity. Partial directed coherence (PDC) analysis of LFP connectivity between L4 and L2/3 under different conditions: (**A**) control (NaCl, *i.p.*); (**B**) low-dose ATM (3 mg/kg, *i.p.*); and (**C**) high-dose ATM (10 mg/kg, *i.p.*). PDC values are presented for OFF (black screen) and ON ([G + N]) epochs across early, intermediate, and late recording phases. Dotted lines represent surrogate PDC values, which preserve the amplitude spectrum and distribution while randomizing phase. This method tests the null hypothesis that observed PDC values arise from linear, stationary processes, helping distinguish genuine directed interactions from spurious effects. Statistical differences between PDC(L4 → L2/3) and PDC(L2/3 → L4) are depicted as blue squares along the lower edge of each panel.

Directional connectivity between cortical layers was assessed under control conditions (saline injection) and following low and high doses of ATM (Figure 3). Each panel in Figure 3 displays frequency-resolved partial directed coherence (PDC) curves, representing the strength and direction of functional interactions from L4 → L2/3 and L2/3 → L4 (y-axis) across frequencies (x-axis). Solid lines depict the observed PDC values, while the lower dotted curves indicate surrogate PDC estimates, serving as a baseline for identifying frequency ranges where connectivity exceeded chance levels. Statistical significance across conditions and frequencies was determined using a Kruskal–Wallis test with Bonferroni-corrected *post hoc* comparisons. Significant PDC components (L4 → L2/3 vs. L2/3 → L4) are marked by blue squares, and non-significant ones by white squares, along the lower edge of each panel. Under control conditions (vehicle injections), we observed a predominant feedforward connectivity pattern characterized by stronger PDC values from L4 to L2/3 when the screen was OFF (black screen): early ~21%, intermediate ~21%, and late ~22% (Figure 3A). This pattern was most pronounced in the higher frequency range (30–50 Hz), aligning with previous findings that gamma oscillations aid sensory encoding and interlaminar communication [36]. During visual stimulation (ON), there was a further increase in L4-to-L2/3 PDC, i.e., early ~30%, intermediate ~26%, and late ~25%, likely due to enhanced cortical excitability and the recruitment of recurrent excitatory circuits [37]. However, administering a low dose of ATM disrupted the typical L4-to-L2/3 connectivity pattern, resulting in reduced PDC values across multiple frequency bands, especially during the intermediate and late phases post-injection (OFF: early ~15%, intermediate ~9%, late ~9%; ON: early ~19%, intermediate ~12%, late ~14%; Figure 3B). Interestingly, this effect was less pronounced with a high dose of ATM (OFF: early ~21%, intermediate ~19%, late ~14%; ON: early ~22%, intermediate ~17%, late ~15%; Figure 3C). These findings indicate that increased activation of adrenergic receptors, due to higher levels of endogenous NE, modulates cortical connectivity [15]. By disrupting normal interlaminar communication in V1, ATM likely weakens feedforward actions from L4 to L2/3, affecting the efficiency of visual processing. This aligns with previous studies showing that activity decorrelation enhances feedforward processing across cortical layers [38,39,40].

### 3.4. Nonlinear Relationship Between Visual Discrimination and Noise Contrast

Sensory processing is affected by various sources of variability. External [N], such as random fluctuations in stimulus contrast, can either enhance or impair perception depending on its intensity and interaction with neural processing [5]. Internal [N], stemming from neural variability, can further influence sensory representations and may interact with external noise to shape perceptual performance [1,2,3]. The principle of stochastic resonance (SR) suggests that moderate [N] levels can enhance signal detectability by raising weak stimuli above a perceptual threshold, while excessive [N] disrupts processing, leading to impaired performance [4,41].

**Figure 4:**
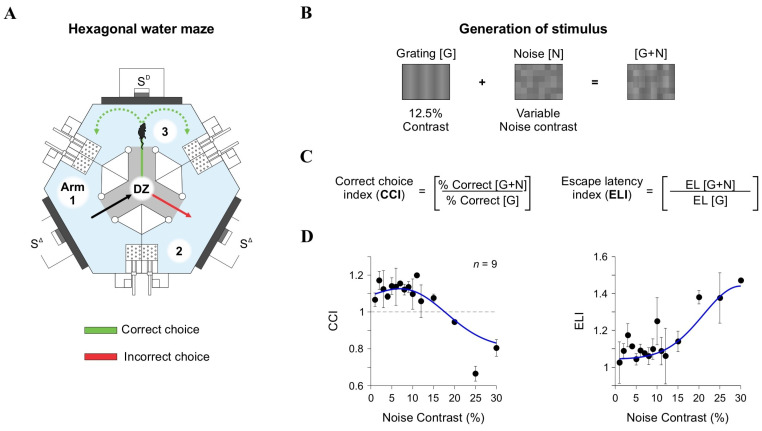
Behavioral task and visual discrimination indices. (**A**) Schematic of the hexagonal water maze, where mice associate a visual stimulus ([G + N]) with an escape platform. Arms of the pool are numbered to facilitate descriptions. (**B**) Stimuli used for behavioral experiments generated identically to those in LFP recordings. (**C**) The Correct Choice Index (CCI) and Escape Latency Index (ELI) were calculated to quantify discrimination accuracy and response time, respectively. (**D**) CCI and ELI as a function of noise contrast, plotted on a linear contrast scale.

To examine whether SR-like effects influence visual decision-making in mice, we employed an automated two-alternative forced-choice (2AFC) task in a hexagonal water maze (Figure 4A). Mice were trained to associate a visual discriminative stimulus (S^D^) with the presence of an escape platform, enabling us to measure task performance through metrics such as % correct choices, escape latency (EL), swimming path length, and velocity [20]. The task design minimized human intervention, ensuring an objective assessment of visual discrimination behavior. In a previous study, we detailed the task, metrics, and showed how performance relies on V1, as pharmacological inactivation with muscimol or lesions with ibotenic acid strongly reduced correct choice behavior [20].

To assess the impact of external noise ([N]) on visual discrimination, we varied [N] contrast from 0% to 30% and added it to gratings [G] with a low contrast of 12.5% (Figure 4B). We then measured the Correct Choice Index (CCI) by comparing correct choices in [G + N] trials to those in [G]-only trials (upper panels in Figure 4C,D). Our findings revealed a nonlinear, inverted U-shaped relationship between CCI and [N] contrast. At moderate [N] levels (7% and 12%), discrimination improved, suggesting that low [N] levels facilitated the detection of weak signals. However, at higher [N] contrasts (20% and 30%), performance declined, supporting the notion that excessive noise hinders perceptual accuracy [5,42]. By fitting the CCI data with a parametric model comprising the sum of a logistic curve and a Gaussian distribution (blue curves in Figure 4D), we confirmed the inverted U-shaped relationship between CCI and [N] contrast. We also analyzed the escape latency index (ELI) as an additional measure of task performance. ELI mirrored the CCI results, displaying the lowest values at moderate [N] contrasts and higher values at elevated [N] levels (lower panels in Figure 4D). These results indicate that intermediate [N] levels not only improved decision accuracy but also enhanced response efficiency, further supporting the notion of an SR-like pattern in behavioral responses from our mice.

### 3.5. Noradrenergic Modulation of Visual Discrimination Across Varying Levels of Visual Noise

Neuromodulators like dopamine and NE play a crucial role in visual perception by regulating cortical excitability and decision-making. They modulate contrast sensitivity, gain control, and SNR in V1, influencing the balance between exploratory and exploitative behaviors in decision-making tasks [14,15,43,44]. We examined how systemic activation of adrenergic receptors affected behavioral performance under different levels of visual [N]. We administered intraperitoneal (*i.p.*) injections of clonidine (CLO), an α_2_-adrenergic receptor agonist that suppresses NE release, or atomoxetine (ATM), a norepinephrine reuptake inhibitor that increases synaptic NE availability. As a control, we used saline (NaCl) injections to assess potential nonspecific effects of the injection procedure. Mice were tested in the 2AFC visual discrimination task under three [N] contrast conditions: 0% (no noise), 7% (moderate noise, optimal for enhancing discrimination), and 30% (high noise, which impaired performance). To evaluate behavioral effects, we measured the percentage of correct responses as an overall performance metric and used the CCI, and ELI as main metrics to quantify the impact of [N] on discrimination accuracy. Additionally, we evaluated choice and EL variance to assess the consistency of decision-making and examined variance in laterality (not illustrated) to determine the consistency of responses regarding the side of the escape platform [21,45].

**Figure 5:**
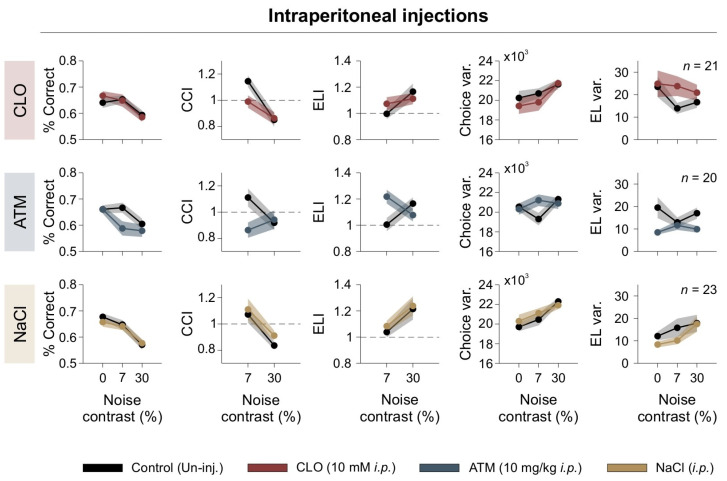
Systemic adrenergic modulation alters visual discrimination. Behavioral performance under systemic adrenergic modulation. Panels show percentage of correct responses, CCI, ELI, choice variability, escape latency variability, as functions of noise contrast. Different pharmacological treatments are color-coded, with control (uninjected) conditions shown in black. Dashed line serves as a reference to identify indices that fall above or below unity.

We found that clonidine impaired visual discrimination by disrupting the beneficial effects of 7% [N] contrast on CCI, and increasing side variability (repeated-measures ANOVA test, choice: *F*_1,20_ = 0.055, *p* = 0.82, *η*^2^_G_ = 0.001; CCI: *F*_1,20_ = 1.89, *p* = 0.184, *η*^2^_G_ = 0.049; CCIǀ_7%_: *F*_1,20_ = 7.11, *p* = 0.0015, *η*^2^_G_ = 0.126; Var(Lat): *F*_1,20_ = 10.5, *p* = 0.004, *η*^2^_G_ = 0.043; RT: *F*_1,20_ = 17.2, *p* < 0.001, *η*^2^_G_= 0.118; Figure 5). Clonidine also increased escape latency, suggesting a disruption in cortical signal integration (ELI: *F*_1,20_ = 0.069, *p* = 0.795, *η*^2^_G_ = 0.001; CCI_RTǀ_7%_: *F*_1,20_ = 2.11, *p* = 0.162, *η*^2^_G_ = 0.034; Var(RT): *F*_1,20_ = 0.828, *p* = 0.37, *η*^2^_G_ = 0.020; Var(RT)ǀ_30%_: *F*_1,20_ = 1.57, *p* = 0.225, *η*^2^_G_ = 0.029; *n* = 21; upper panels in Figure 5).

Atomoxetine decreased performance at low and high [N] levels, reducing CCI and increasing choice variability (choice: *F*_1,19_ = 6.97, *p* = 0.016, *η*^2^_G_ = 0.073; CCI: *F*_1,19_ = 3.07, *p* = 0.046, *η*^2^_G_ = 0.086; Var(Lat): *F*_1,19_ = 0.661, *p* = 0.426, *η*^2^_G_ = 0.002; RT: *F*_1,19_ = 16.3, *p* < 0.001, *η*^2^_G_ = 0.076; CCI_RT: *F*_1,19_ = 1.33, *p* = 0.264, *η*^2^_G_ = 0.043; ELIǀ_7%_: *F*_1,19_ = 5.56, *p* = 0.029, *η*^2^_G_ = 0.183; Var(RT): *F*_1,19_ = 8.26, *p* = 0.01, *η*^2^_G_ = 0.121; *n* = 20; middle panels in Figure 5). Interestingly, atomoxetine-induced choice variability was most pronounced at intermediate noise levels, suggesting a nonlinear interaction between adrenergic modulation and sensory noise during decision-making.

In contrast, saline injections had no significant effect on any parameters, confirming that the behavioral changes observed with clonidine and atomoxetine were specific to noradrenergic modulation (repeated-measures ANOVA test, choice: *F*_1,27_ = 0.261, *p* = 0.613, *η*^2^_G_ = 0.003; CCI: *F*_1,27_ = 1.11, *p* = 0.301, *η*^2^_G_= 0.021; Var(Lat): *F*_1,27_ = 0.264, *p* = 0.611, *η*^2^_G_ = 0.00; RT: *F*_1,27_ = 0.098, *p* = 0.756, *η*^2^_G_ = 0.001; ELI: *F*_1,27_ = 0.263, *p* = 0.612, *η*^2^_G_ = 0.005; Var(RT): *F*_1,27_ = 1.12, *p* = 0.30, *η*^2^_G_ = 0.013; *n* = 28; lower panels in Figure 5). These findings reveal the key role of noradrenergic modulation in shaping visual decision-making under noisy conditions, likely affecting the interaction between visual noise and internal neural processing by modulating cortical gain.

### 3.6. Intracortical Adrenergic Modulation of Visual Discrimination

To investigate the acute effects of adrenergic modulation on visual processing while minimizing systemic influences, we conducted bilateral intracortical micro-infusions directly into V1 of mice [19,20]. This approach enabled direct pharmacological action on V1 without influencing peripheral systems. We tested two concentrations of NE (NE-low: 0.025 mM; NE-high: 100 mM) and a combination of adrenergic antagonists targeting all adrenergic receptor subtypes (PPY: prazosin + propranolol + yohimbine). Behavioral performance was assessed across [N] contrast conditions of 0%, 7%, and 30%, using the same behavioral metrics as in the previous section.

**Figure 6:**
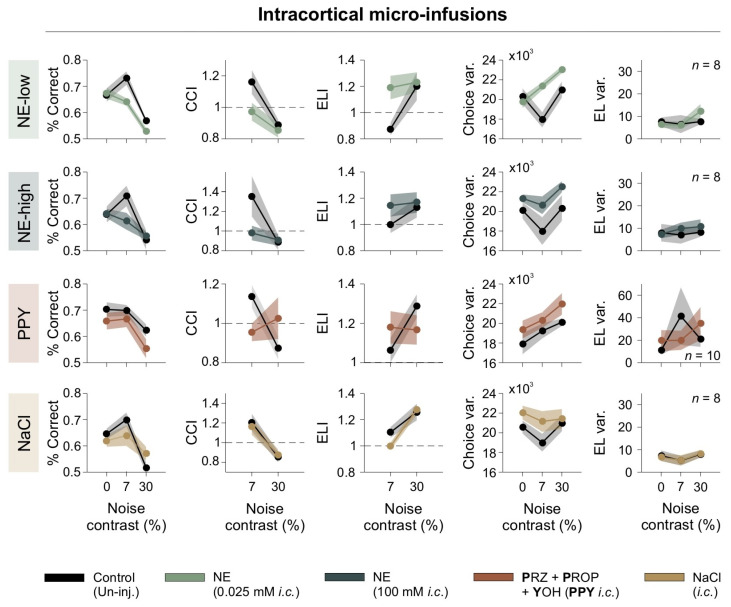
Intracortical adrenergic modulation impairs discrimination. Behavioral effects of intracortical adrenergic modulation. Panels depict percentage of correct responses, CCI, ELI, choice variability, escape latency variability, as functions of noise contrast. Treatments are color-coded, with control (uninjected) conditions shown in black. Dashed line serves as a reference to identify indices that fall above or below unity.

The micro-infusion of low-dose NE (NE-low) negatively affected performance by reducing both the %correct responses and the CCI, particularly at a 7% [N] contrast. It also increased choice variability (repeated-measures ANOVA test, choice: *F*_1,7_ = 11.9, *p* = 0.011, *η*^2^_G_ = 0.305; CCI: *F*_1,7_ = 5.98, *p* = 0.044, *η*^2^_G_ = 0.166; Var(Lat): *F*_1,7_ = 0313, *p* = 0.865, *η*^2^_G_ = 0.00; RT: *F*_1,7_ = 0.195, *p* = 0.672, *η*^2^_G_ = 0.009; ELI: *F*_1,7_ = 7.26, *p* = 0.031, *η*^2^_G_ = 0.275; Var(RT): *F*_1,7_ = 0.242, *p* = 0.638, *η*^2^_G_ = 0.009; *n* = 8; first row of Figure 6). High-dose NE (NE-high) also impaired discrimination, causing a similar reduction in performance at 7% noise and an increase in choice variability (*F*_1,7_ = 1.97, *p* = 0.203, *η*^2^_G_ = 0.097; CCI: *F*_1,7_ = 2.54, *p* = 0.155, *η*^2^_G_= 0.162; CCIǀ_7%_: *F*_1,7_ = 3.67, *p* = 0.009, *η*^2^_G_ = 0.232; Var(Lat): *F*_1,7_ = 1.32, *p* = 0.288, *η*^2^_G_ = 0.002; RT: *F*_1,7_ = 1.20, *p* = 0.310, *η*^2^_G_ = 0.036; ELI: *F*_1,7_ = 1.22, *p* = 0.307, *η*^2^_G_ = 0.032; ELIǀ_7%_: *F*_1,7_ = 2.60, *p* = 0.151, *η*^2^_G_ = 0.146; Var(RT): *F*_1,7_ = 0.439, *p* = 0.53, *η*^2^_G_ = 0.011; *n* = 8; second row of Figure 6). These results indicate that low and high intracortical NE infusions disrupted discriminative behavior and increased choice variability.

To further investigate the role of adrenergic receptors in visual discrimination, we included an additional condition, i.e., PPY, which involves a complete blockade of adrenergic receptors, thereby preventing activation by endogenous norepinephrine (NE). The aim of testing PPY was to determine whether blocking all adrenergic receptor subtypes impaired visual performance, thereby evaluating the contribution of endogenous NE to visual discrimination. Notably, full adrenergic receptor blockade led to a reduction in correct choices and CCI at 7% [N] contrast (choice: *F*_1,9_ = 14.9, *p* = 0.004, *η*^2^_G_ = 0.181; CCI: *F*_1,9_ = 0.130, *p* = 0.727, *η*^2^_G_ = 0.006; ELIǀ_7%_: *F*_1,9_ = 21.9, *p* = 0.001, *η*^2^_G_ = 0.222; Var(Lat): *F*_1,9_ = 1.61, *p* = 0.236, *η*^2^_G_ = 0.005; RT: *F*_1,9_ = 2.93, *p* = 0.121, *η*^2^_G_ = 0.072; ELI: *F*_1,9_ = 0.001, *p* = 0.989, *η*^2^_G_ = 0.000; Var(RT): *F*_1,9_ = 1.84, *p* = 0.208, *η*^2^_G_ = 0.082; *n* = 10; third row of Figure 6). These results suggest that endogenous NE signaling is essential for optimal task performance.

In contrast, saline micro-infusions did not significantly impact any behavioral parameter, confirming that the observed changes were specifically due to adrenergic modulation (choice: *F*_1,7_ = 0.267, *p* = 0.621, *η*^2^_G_ = 0.020; CCI: *F*_1,7_ = 0.094, *p* = 0.767, *η*^2^_G_ = 0.011; Var(Lat): *F*_1,7_ = 3.29, *p* = 0.112, *η*^2^_G_ = 0.00; RT: *F*_1,7_ = 2.78, *p* = 0.139, *η*^2^_G_ = 0.036; CCI_RT: *F*_1,7_ = 1.63, *p* = 0.243, *η*^2^_G_ = 0.125; Var(RT): *F*_1,7_ = 0.007, *p* = 0.97, *η*^2^_G_ = 0.000; *n* = 8; fourth row of Figure 6). These findings demonstrate that NE significantly impacts visual discrimination and underscore the importance of normal adrenergic receptor activity in maintaining performance.

## 4. Discussion

We explored how visual noise ([N]) affects the cortical processing of low-contrast stimuli in V1 and how noradrenergic (NE) modulation influences these neural responses. Specifically, we assessed the impact of visual noise on neural activity and behavior and whether NE modulates the SNR in V1. We conducted in vivo electrophysiological recordings in sedated mice, measuring LFPs in L2/3 and L4 during visual stimulation with gratings ([G]) and varying [N] contrast. Additionally, we performed behavioral experiments in freely moving mice using a hexagonal water maze-based visual discrimination task, quantifying the correct choice index (CCI) and escape latency (EL) across different [N] contrast levels [20].

Our findings demonstrate that both neural activity and behavior exhibited peak SNR response patterns at low [N] contrast levels consistent with stochastic resonance (SR). This well-documented sensory phenomenon suggests that moderate noise levels enhance neural responses and perceptual accuracy, while excessive noise disrupts both [4,41]. The LFP power spectra in V1 mirrored this nonlinear pattern, showing that cortical responses to sensory stimuli were optimal at intermediate noise levels (7–12%), whereas higher noise levels degraded sensory processing. This SR-like effect aligns with previous research indicating that moderate background noise improves detection thresholds in vision, audition, and somatosensation [5,46,47]. In visual perception, SR enhances contrast and motion discrimination [4,5,42], supporting the notion that the visual system exploits noise to amplify weak stimuli under specific conditions. Our behavioral data reinforce this hypothesis: mice exhibited optimal discrimination at intermediate noise levels (7–12%) but showed impaired performance at higher noise levels (>20%). While the mechanisms underlying SR effects in V1 remain under investigation, our results suggest they may stem from contrast-dependent cortical gain control. In this model, moderate noise modulates neural excitability, boosting weak signals, whereas excessive noise disrupts sensory processing.

The balance between external and internal noise is crucial in shaping perceptual performance. External noise encompasses stimulus-dependent fluctuations like contrast variation or visual clutter, while internal noise arises from neural variability within the visual system. Contrast processing in V1 is governed by the interplay between feedforward thalamocortical inputs and recurrent intracortical interactions [13]. At low contrast, lateral cortical connections dominate, integrating spatially distributed signals to enhance detection. However, as contrast increases, direct feedforward inputs from the dorsal lateral geniculate nucleus (dLGN) become more prominent, sharpening stimulus representation [10,11]. This shift from lateral to feedforward processing [12] may account for the contrast-dependent modulation of spectral activity observed in our LFP recordings. Layer-specific dynamics in V1 support this interpretation. For example, narrowband gamma suppression in L4, the primary recipient of thalamic inputs, suggests that increasing contrast diminishes recurrent cortical processing while enhancing direct sensory-driven responses [48]. Moderate noise optimizes contrast encoding, while adrenergic modulation flattens the SNR curve, indicating that neuromodulators actively regulate how V1 circuits integrate sensory information. Behaviorally, adding an optimal level of noise to the visual cortex can enhance the quality of subthreshold signals, improving the rate of evidence accumulation during decision-making tasks [46]. The enhancement of evidence accumulation through stochastic resonance can lead to improved decision-making when stimuli are near the perceptual threshold but not when they are well below or above it [46]. The visual cortex (V1) and frontal cortex (MOs) play crucial roles in processing visual information and making decisions [49].

A key aspect of our study was exploring how NE signaling affected noise processing in V1. Adrenergic receptors (ARs), i.e., α_1_-, α_2_-, and β-ARs, are known to regulate cortical excitability and gain control mechanisms [15,19]. Our findings show that adrenergic modulation impacts cortical responses to visual noise. First, systemic administration of atomoxetine (ATM), a norepinephrine reuptake inhibitor, disrupted the optimal SNR curve in a dose-dependent manner. High doses of ATM flattened the SR function, suggesting that excessive NE increased internal noise, compromising sensory representations. Second, clonidine, an α_2_-adrenergic agonist that reduces NE release, impaired discrimination at moderate noise levels, highlighting that NE levels are necessary for tuning cortical gain in response to external variability. Third, intracortical micro-infusions of NE impaired visual discrimination at both low and high doses. These findings align with models of cortical gain control, where excessive NE destabilizes neural computations, introducing uncontrolled variability [15,19]. Neuromodulatory systems such as NE and acetylcholine regulate cortical state transitions, maintaining a balance between stability and flexibility in response to sensory uncertainty [49]. A limitation of our study is that ATM not only increases norepinephrine levels but also affects dopamine, acts as an NMDAR antagonist, and may influence kappa-opioid receptors [50]. These additional effects could alter glutamatergic transmission and confound the interpretation of our results.

The interaction between internal (neuronal variability within V1) and external (stimulus-related variability) noise could obey different principles. In low-external-noise conditions, internal noise could become a limiting factor in perception as it introduces uncertainty in the neural representation of the stimulus. Conversely, when external noise is high, the visual system may engage noise-filtering mechanisms, such as contrast gain control, divisive normalization, or attentional modulation, to optimize signal extraction. Furthermore, when external noise is present, V1 neurons may operate in a SR regime, enhancing the detection of weak signals by pushing subthreshold inputs to suprathreshold levels.

Neural variability is not just a source of noise; it is a fundamental feature of flexible cortical computation. Rather than degrading perception, background noise and response variability could enhance the computational range of cortical networks [51,52]. Several pieces of evidence support this view. Synaptic noise influences neuronal excitability, affecting how subthreshold fluctuations lead to action potential generation. Trial-to-trial variability in cortical responses can predict perceptual decisions, indicating that neural activity fluctuations directly impact behavior. Gamma-band synchronization (~30–80 Hz) in V1, modulated by stimulus contrast, aids feedforward sensory processing and decision-making [11,53]. Our findings suggest that NE modulates cortical variability, consistent with models suggesting that extreme NE levels destabilize sensory computations, disrupting the balance between signal enhancement and noise suppression [15,33].

Oscillatory activity is also a fundamental mechanism that structures neural communication, with gamma-band synchronization playing a crucial role in sensory processing [54]. Gamma coherence is influenced by stimulus properties, behavioral state, and neuromodulatory inputs, providing a dynamic mechanism for optimizing cortical computations [53,55]. The interaction between neural variability and oscillatory coherence determines the reliability of sensory representations, balancing perceptual stability with flexibility [56]. Neuromodulators such as NE shape oscillatory coherence, affecting cognitive control and adaptive behavior [57]. Noradrenergic activity is linked to reduced neural noise, stabilizing task-relevant representations in decision-making [58]. Similarly, cholinergic modulation enhances response precision, highlighting the role of neuromodulatory systems in sensory sharpening and cognitive stability [59]. In V1, NE modulates gamma synchronization in L2/3 and low-frequency oscillations in L4. Under high-contrast conditions, L2/3 shows increased gamma activity, supporting feedforward processing and feature encoding, while L4 exhibits enhanced low-frequency oscillations (alpha and beta bands), reflecting recurrent processing and feedback integration [60,61]. Adrenergic receptor activation further modulates these laminar responses: clonidine suppresses gamma power in L2/3 while increasing low-frequency activity in L4, suggesting enhanced top-down inhibitory control, whereas atomoxetine increases gamma synchronization in L2/3 while reducing low-frequency power in L4, indicating enhanced cortical excitability [15,59].

Our partial directed coherence (PDC) analysis revealed how visual noise and adrenergic modulation impact interlaminar connectivity in V1. Under control conditions, we observed a predominant feedforward influence from L4 to L2/3, consistent with hierarchical sensory processing models. This pattern aligns with findings that L4 receives thalamocortical input, which is relayed to L2/3 for further integration [36]. Gamma-band activity in L4 reflects thalamocortical drive, while L2/3 exhibits enhanced gamma coherence, supporting its role in cortical integration and higher-order processing [60,61]. However, adrenergic modulation significantly altered interlaminar connectivity. High doses of atomoxetine (ATM) disrupted L4 to L2/3 PDC, suggesting that excess NE weakens feedforward processing by increasing neural variability. This aligns with research showing that adrenergic modulation shifts cortical dynamics from stable, stimulus-driven states to high-variability, exploratory states [38]. The disruption of L4 to L2/3 connectivity indicates that excessive NE may reduce sensory reliability by impairing hierarchical signal propagation.

To assess the impact of noise contrast on visual discrimination performance, we introduced varying noise levels (0%, 7%, 12%, 20%, and 30%) into low-contrast visual gratings and measured behavioral responses using the CCI. This metric, which calculates the proportion of correct choices during trials with noise ([G + N]) relative to noise-free conditions ([G]), provided a quantitative measure of discrimination accuracy. Our results revealed a nonlinear, stochastic resonance-like relationship between noise contrast and CCI. At moderate noise contrasts (7% and 12%), mice showed improved discrimination, suggesting that moderate noise facilitated weak signal detection. However, higher noise contrasts (20% and 30%) impaired performance, likely due to excessive interference with the visual signal. These findings align with stochastic resonance (SR) models, which propose that optimal noise levels enhance perception, while excessive noise disrupts it [5,42].

A crucial aspect of our study was the parallel between neural and behavioral responses to visual noise. The inverted U-shaped function of CCI as a function of noise contrast mirrored, to some degree, the neural SNR patterns observed in V1, supporting the hypothesis that stochastic resonance mechanisms in cortical circuits directly influence perceptual decision-making. These results are consistent with prior research in both humans and rodents, showing that moderate noise improves motion discrimination while excessive noise impairs it [5]. Beyond the effects of external noise, we examined the role of adrenergic modulation in regulating internal cortical noise. Our behavioral results highlighted key effects of adrenergic manipulation: Clonidine (an α_2_-adrenergic agonist that suppresses NE release) impaired discrimination, particularly at moderate noise levels (7% contrast), confirming that balanced NE levels are necessary for tuning cortical gain to external variability. Atomoxetine (an NE reuptake inhibitor) disrupted discrimination performance across all noise levels, reducing CCI and increasing response variability, especially at intermediate noise levels. Intracortical NE micro-infusions at both low (NE-low) and high doses (NE-high) impaired discrimination performance, reducing CCI at 7% noise contrast and increasing trial-to-trial choice variability.

Adrenergic modulation also influenced decision-making beyond direct sensory processing. Both clonidine and atomoxetine increased choice variability and escape latencies, suggesting impaired decision consistency and prolonged response times. Clonidine increased response variability at moderate noise levels, disrupting the beneficial effects of noise on discrimination accuracy, while increased escape latencies suggested impaired cortical integration, leading to slower decision-making. Atomoxetine increased response variability at intermediate noise levels, reinforcing that excessive NE disrupts stable decision-making. These findings align with studies demonstrating that NE stabilizes perceptual decisions under uncertainty [62], suggesting that noradrenergic signaling plays a pivotal role in determining whether sensory processing remains stable or shifts toward an exploratory state based on environmental demands. An interesting observation regarding our cortical micro-infusions is that there were similar effects by NE and adrenergic antagonists on visual discrimination performance. Several potential mechanisms could explain this outcome. For instance, NE has a well-documented dose-dependent effect on cortical processing. Both excessive and insufficient NE levels could impair performance, while intermediate levels optimize sensory processing. Alternatively, excessive NE could have led to hyper-excitability or disrupted local inhibition, impairing sensory discrimination in a way that mirrors the effects of NE blockade, which would reduce excitability and signal transmission efficacy. Moreover, the observed behavioral outcomes may not solely reflect local V1 processing but also interactions with higher-order visual or decision-making areas (e.g., prefrontal cortex, superior colliculus).

While our pharmacological manipulations specifically targeted the primary visual cortex (V1), and our conclusions regarding adrenergic modulation and the processing of visual contrast noise are centered on local V1 circuits, it is important to recognize that decision-making in mice emerges from the coordinated activity of broader neural networks. V1 plays a key role in encoding visual evidence and contributes to early sensory stages of decision formation. However, downstream areas such as the parietal cortex are critically involved in integrating this sensory information over time, supporting both the accumulation of evidence and short-term memory-dependent components of perceptual decision-making [63,64]. Other brain regions, including the anterior insular cortex (AIC), also modulate decision strategies, especially under uncertainty or emotional stress. In particular, the AIC has been implicated in regulating risk-based decisions, and its activity is modulated by factors such as hormonal state, highlighting its role in adaptive behavioral control [65].

Effective decision-making in dynamic environments depends on the brain’s ability to integrate sensory evidence with contextual information while continuously adjusting strategies based on changing input. A central challenge in this process involves distinguishing between reducible and irreducible uncertainty—a key concept in Bayesian inference models. These models propose that decision-makers optimize behavior by selectively filtering out irrelevant noise and prioritizing informative signals to update internal beliefs and guide future choices [66]. Our findings support this theoretical framework by demonstrating that noradrenergic modulation alters cortical responses to visual stimuli in a noise-dependent manner, influencing how uncertainty is managed during perceptual decision-making.

Specifically, we found that systemic administration of atomoxetine, a norepinephrine (NE) reuptake inhibitor, increased behavioral variability and impaired correct choice rates under moderate noise conditions. In contrast, clonidine, which suppresses NE release, elevated trial-to-trial variability and disrupted sensory reliability. These results suggest that NE contributes to perceptual stability by modulating internal cortical noise and signal processing, especially when external sensory conditions are ambiguous. Under low-noise conditions, NE reuptake inhibition improved discrimination, consistent with its proposed role in enhancing sensory precision and stabilizing decision policies.

Beyond sensory encoding, our results raise the possibility that NE influences higher-order cognitive processes, such as confidence estimation and belief updating. Confidence in prior decisions has been shown to shape future responses, with high-confidence judgments leading to more conservative or cautious behavior [67]. An adrenergic receptor blockade may disrupt this process, interfering with dynamic adjustments in decision thresholds. Given that NE also regulates behavioral flexibility and motivation [68], its broader impact on adaptive behavior likely includes metacognitive domains, such as strategy selection and self-monitoring.

Neural variability plays a central role in shaping decision-making strategies. In rodents, stable activity patterns in the medial frontal cortex (MFC) have been associated with consistent choice behavior across trials, suggesting that network stability supports individual decision policies [69]. Given that NE modulates cortical excitability and variability, it may influence how animals navigate the exploration–exploitation trade-off, i.e., adjusting between known strategies and novel options in response to changing environments. This hypothesis could be tested using behavioral paradigms that manipulate uncertainty or reward dynamics. An important direction for future research is to determine whether adrenergic modulation differentially affects learning across timescales. Effective decision-making requires integrating information over both short and long horizons, particularly in non-stationary contexts [70]. Sequential decision-making tasks with controlled uncertainty could help reveal whether NE influences rapid adaptation, gradual belief updating, or both.

## Data Availability

The original data presented in the study are openly available in https://osf.io/4w28k/?view_only=866411a7c9aa4149943d969c652e2b68 (accessed on 19 March 2025).

## Abbreviations

CCI	Correct Choice Index
ELI	Escape Latency Index
ACh	Acetylcholine
ATM	Atomoxetine
AUC	Area Under the Curve
CLO	Clonidine
dLGN	Dorsal Lateral Geniculate Nucleus
EL	Escape Latency
FPS	Frames Per Second
GABA	Gamma-Aminobutyric Acid
*i.c.*	Intracortical
*i.p.*	Intraperitoneal
KW	Kruskal–Wallis Test
L2/3	Layer 2/3 of Cortex
L4	Layer 4 of Cortex
LC	Locus Coeruleus
LFP	Local Field Potential
NE	Norepinephrine
PDC	Partial Directed Coherence
PPY	Prazosin + Propranolol + Yohimbine
RM-ANOVA	Repeated-Measures ANOVA
SEM	Standard Error of the Mean
SNR	Signal-to-Noise Ratio
SR	Stochastic Resonance
V1	Primary Visual Cortex
